# CNS Structural Anomalies in Iranian Children with Global Developmental Delay

**Published:** 2013

**Authors:** Gholam Reza ZAMANI, Reza SHERVIN-BADV, Ali NIKSIRAT, Houman ALIZADEH

**Affiliations:** 1 Associate Professor of Pediatric Neurology, Children’s Medical Center, Tehran University of Medical Sciences, Tehran, Iran; 2 Assistant Professor of Pediatric Neurology, Department of Pediatric Neurology, Zanjan University of Medical Sciences, Zanjan, Iran; 3 3.General Physician, Legal Medicine Research Center, Legal Medicine Organization, Tehran, Iran; 4 Neuroradiologist, Children’s Medical Center, Tehran University of Medical Sciences, Tehran, Iran

**Keywords:** Developmental delay, CNS developmental anomalies, Etiology, Neuroimaging

## Abstract

**Objective:**

Central Nervous system (CNS) malformations are one of the most important causes of global developmental delay (GDD) in Children. About one percent of infants with GDD have an inherited metabolic disorder and 3-10 percent have a chromosomal disorder. This study aimed to survey the frequency of brain structural anomalies and their subtypes among the variety of etiologic factors in children with GDD in our patients.

**Materials & Methods:**

This study used the results of neuroimaging studies [unenhanced brain Magnetic Resonance Imaging (MRI)] of all children who had been referred for evaluation of GDD to outpatient Clinic of Pediatric neurology at Children’s Medical Center affiliated to Tehran University of Medical Science between September 2009 and September 2010.

**Results:**

In this study, unenhanced brain MRI was performed on 405 children, of which 80 cases (20 percent) had brain structural anomalies. In 8.7 percent of the cases, previous history of brain structural disorders existed in other children of the family and 20 percent of mothers had inadequate consumption of folate during pregnancy.

**Conclusion:**

Based on the results of this study, unenhanced cranial MRI seems to be a fundamental part of evaluation in all children with GDD. Adequate folate consumption as prophylaxis as well as genetic counseling can be worthy for high-risk mothers who have previous history of CNS anomaly or miscarriage to avoid repeated CNS anomalies in their next pregnancies.

## Introduction

Delay in achievement of developmental milestones is one of the most common problems seen by child neurologists. The problem affects 1-3% of the population (1, 2). Developmental Delay can be divided into two subsets, isolated developmental delay (IDD) and global developmental delay (GDD). In IDD only one aspect of development in either motor or cognitive domain is involved, while in GDD a significant delay is seen in two or more developmental domains (gross/fine motor, cognitive, speech/language, personal/social behavior or activities of daily living) (3,4). Variable causes of GDD have been recognized, such as brain structural anomalies, chromosomal disturbances, inborn errors of metabolism, intrauterine infections and perinatal insults (1,5).

Thorough history and neurological examination are key points to produce a formulation for approach and investigation of such cases appropriately. Brain structural anomalies occur during embryonic development. These disorders can be caused by external factors (teratogenic or infectious agents), internal or genetic factors, and biochemical defects in mother, or interaction between these factors (5,6). The incidence of brain malformations has been estimated to be approximately 3.32 per 1000 and the prevalence is approximately 2.21 per 1000 at age 14 years based on the studies of a 1-year birth cohort from northern Finland in 1986. (7).

These are now much higher rates than were recognized in that era before advanced MRI being easy accessible and before recent increase in surgical treatment for hydrocephalus and epilepsy (8-10). Brain structural anomalies are responsible for 13 percents of infants’ death (11,12). Brain structural anomalies are also the most common cause of symptomatic epilepsy in children and significant cause of developmental delay. Despite all advances in neuroimaging, neurometabolic investigations and molecular genetics, etiology of CNS structural anomalies is unknown in more than 60 percent of patients [13]. This study was performed to survey frequency of brain structural anomalies and their subtypes among the variety of etiologic factors in children with global developmental delay (GDD) in our patients.

## Materials & Methods

A total of 405 children with GDD referred to Children’s Medical Center in Tehran from September 2009 to September 2010 were recruited in this study. Brain imaging (unenhanced brain MRI) was done for all of them. Neuroimages were reviewed by a neuroradiologist and pediatric neurologist. Findings were recorded in a questionnaire designed to record the clinical, demographic, metabolic and radiologic findings of the patients with brain structural disorder.Data was analyzed by SPSS 16 statistical software. The study was approval by the Ethics Committee at Tehran University of Medical Sciences.

## Results

Of 405 children with GDD, 80 patients (20%) had brain developmental anomalies. These cases were 38 boys and 42 girls with mean age of 21.5 months. The mean age of mothers during pregnancy was 24.95 years and the mean age of fathers (during mothers pregnancy) was 31.5 years. Our Investigation demonstrated Corpus callosum agenesis in 15 out of 80 cases, pachygyria complex in 11, cerebellar hypoplasia in 9, lissencephaly in 9, schizencephaly in 7, holoprosencephaly in 7, polymicrogyria in 5, megalencephaly in 4, Dandy– Walker syndrome in 4, primary microcephaly in 2, hemimegalencephaly in 2, Joubert syndrome in 2, one periventricular heterotopia, one Chiari type 1 malformation, and one cerebellar vermis hypoplasia. Gender distribution of patients was shown in the table 1. 31 percent of patients had history of seizure that schizencephaly and polymicrogyria were more frequent (71% and 60% of this group, respectively). In 8.7% of cases, previous history of brain structural disorders was present in other children of the family. 36 percent of parents were close relatives (cousin – cousin). 20 percent of mothers during pregnancy had inadequate consumption of folate according to WHO guideline. 28 percent of mothers in holoprosencephaly group had a history of abortion. 12.5 percent of mothers had history of fever, hypertension, diabetes, or blunt trauma during pregnancy.

## Discussion

GDD has a wide range of etiology and is an important cause of referral to pediatricians and pediatric neurologists (14). Selective investigations are useful in determining the cause, but the cornerstone of the diagnostic process is careful clinical examination. For investigation of GDD so many works, such as extended neurometabolic studies, molecular genetic tests, neuroimaging, visual and hearing examinations besides history taking and physical examination, may be needed (15). Approximately one percent of infants with GDD have inherited metabolic disease and about 3.5-10 percent have chromosomal disorders and brain developmental anomalies accounts for up to 13 percent of morbidities in some studies (4,11,12). In our study, 80 out of 405 children with GDD had brain structural disorder (20 percent) which seems to be a significant rate. The patients’ mean age was 21.5 months which seems to be a little late for early detection and early intervention. The least mean age at referral time was in patients with holoprosencephaly who were symptomatic due to earlier presentation of clinical symptoms pushing them to seek for medical assessment and the maximum age of referral was in those with vermis hypoplasia. The mean age of mothers was 24.95 years and in none of the groups the mothers mean age was more than 35 years. The mean age of the fathers was 41.2 years, among which the minimum age was in a case with hemimegalencephaly (24 years) and in another case with vermis hypoplasia (22 years). The maximum age among fathers was in a case with microcephaly (38 years). However, in this study we did not detect any correlation between the parents’ age and developmental anomalies of brain, indicating that other factors besides mother’s or father’s age may be relevant in this regard. Our data showed that 20 percents of mothers had inadequate consumption of folate during pregnancy while according to WHO guideline, pregnant women should take a minimum of 400 micrograms of folic acid at least one month before the conception up to 20th week of pregnancy (or to the end of 5th month of pregnancy) or even start much earlier, preferably 3 months with much higher doses before conception in those with previous history of dysraphism (16). Adequate folate consumption is a simple prophylactic recommendation that must not be missed. Our results showed 8.7% of the cases had previous delivery of brain structural anomalies, as in two cases with Joubert syndrome which is a disorder with autosomal recessive inheritance. Past history of abortion in previous pregnancies existed in some of the cases, mainly in those with holoprosencephaly that is justifiable for high incidence chromosomal abnormalities accompanied with holoprosencephaly. The noteworthy point was that none of the cases who had previous history of CNS anomaly or miscarriage, had not received any notice about genetic counseling for their next pregnancy. 31 percent of our patients in this study had a history of seizure that was mostly in those with schizencephaly and the other migrational anomalies that can be explained due to involvement of cerebral cortex in these disorders. 8.7 percent of mothers had risk factors such as fever, diabetes, hypertension, UTI, or blunt trauma during their pregnancy.

**Table 1:** Gender Distribution of the Patients with Global Developmental Delay

**Brain Structural Anomaly**	**Gender of patients Number (percent)**	
	**Male**	**Female**
Schizencephaly (7)	2 (28.6)	5 (71.4)
Holoprosencephaly(7)	2 (28.6)	5 (71.4)
Agenesis of corpus callosum (15)	5 (33.3)	10 (66.7)
Mircocephaly (2)	1 (50)	1 (50)
Megaencephaly (4)	4 (100)	0
Hemimegalencephaly (2)	1 (50)	1 (50)
Periventricular heterotopia(1)	0 (0)	1 (100)
Lissencephaly (9)	8 (88.9)	1 (11.1)
Pachygyria (11)	7 (63.6)	4 (36.4)
Polymicrogyria(5)	2.(40)	3 (60)
Dandy-Walker syndrome (4)	3 (75)	1 (25)
Joubert syndrome (2)	1 (50)	1 (50)
Cerebellar hypoplasia (9)	2 (22.2)	7 (77.8)
Chiari type 1 (1)	0 (0)	1 (100)
Vermis hypoplasia (1)	0 (0)	1 (100)
Total	38 (48)	42 (52)

**In conclusion, **based on the previous studies, inherited metabolic disorders include one percent of the etiologic factors, chromosomal abnormalities about 3-10 percent and brain developmental anomalies explain up to 13 percent of morbidities in some studies (11,12). According to our study, brain structural abnormalities were responsible for GDD in twenty percents of patients, so performing an advance MR imaging to detect brain structural anomalies in addition to laboratory findings or molecular genetic studies is highly recommended as a logical part of diagnostic approach for evaluation of these children. On the other hand, adequate folate consumption as prophylaxis is recommended to all mothers with previous CNS anomalies and finally genetic counseling can be beneficial for high risk mothers who have previous history of CNS anomaly or miscarriage to avoid repeated CNS anomalies in their next pregnancies. According to these findings of this study, further studies with larger samples are recommended. 
